# Degradation of Extracellular Matrix by Matrix Metalloproteinase 2 Is Essential for the Establishment of the Blood-Brain Barrier in *Drosophila*

**DOI:** 10.1016/j.isci.2019.05.027

**Published:** 2019-05-27

**Authors:** Hiroshi Kanda, Rieko Shimamura, Michiko Koizumi-Kitajima, Hideyuki Okano

**Affiliations:** 1 Department of Physiology, Keio University School of Medicine, 35 Shinanomachi, Shinjuku-ku, Tokyo 160-8582, Japan

**Keywords:** Biological Sciences, Molecular Biology, Cell Biology

## Abstract

The blood-brain barrier (BBB) is an essential system that isolates the central nervous system from the internal environment. Increasing evidence has begun to reveal the molecules that are required for BBB integrity. However, how these components are regulated remains unclear. Here we report that a matrix metalloproteinase, Mmp2, is essential for the establishment of the BBB in *Drosophila*. In the absence of *mmp2*, the BBB becomes leaky, which allows the tracer to penetrate the brain. Moreover, the expression pattern of a junctional component, Neuroglian, is altered. We also find that the regulation of the amounts of particular extracellular matrix components is critical for BBB establishment. Furthermore, the process of mesenchymal-epithelial transition of BBB-forming cells is perturbed in the absence of Mmp2. These data indicate that the presence of Mmp(s), which is typically considered to be a risk factor for BBB degradation, is essential for BBB integrity in *Drosophila*.

## Highlights

• Mmp2 is required for the establishment of the blood-brain barrier in *Drosophila*
• Regulation of the amounts of particular ECM components is critical
• Mesenchymal-epithelial transition is impaired in the absence of Mmp2

## Graphical Abstract

**Figure:**
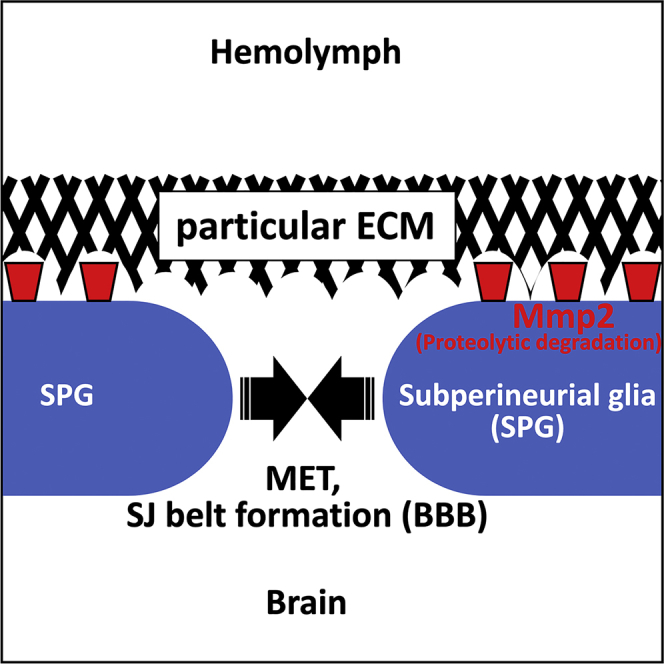

## Introduction

The blood-brain barrier (BBB) is a tissue architecture that regulates the strongly “isolated” microenvironment of the central nervous system (CNS). In the mammalian CNS, the BBB is established by microvascular capillary endothelial cells. There are remarkable differences in the properties of vessels (capillaries) in the brain and periphery in vertebrates. For example, in contrast to leaky vessels in peripheral organs (Mann et al., 1985), the BBB restricts the entry of polar molecules as well as peptides and proteins into the brain (Zloković et al., 1985, Zlokovic et al., 1985, Zlokovic and Apuzzo, 1997). However, neuroactive peptides can still be transported into the brain via specific transporters expressed in the brain endothelium under physiological or pathological conditions (Gögelein and Capek, 1990, Zloković et al., 1987). In addition, several BBB peptide transport mechanisms exist, for example, receptor-mediated, adsorptive-mediated, and carrier-mediated ones, as well as nonspecific passive diffusion (Zlokovic, 1995).

The paracellular permeability of the most hydrophilic molecules from the circulation into the brain is restricted by tight junctions (TJs) between endothelial cells. A growing body of research has identified junctional proteins, as well as intracellular molecules, that regulate the properties of the BBB (Daneman and Prat, 2015). However, how these molecules are regulated remains unresolved. Recently, two groups have reported that pericytes, which ensheathe endothelial cells, are required for BBB formation (Armulik et al., 2010, Daneman et al., 2010), clearly indicating the importance of non-cell-autonomous signaling.

The extracellular environment is often highlighted when the BBB is disrupted in pathological conditions, such as ischemia and brain tumors (Rempe et al., 2016). In these conditions, excess amounts of pro-inflammatory cytokines, as well as matrix metalloproteinases (Mmps), are secreted. Mmps are endopeptidases that are involved in multiple processes, such as tissue formation and dynamic remodeling, in patho- or physiological conditions (Page-McCaw et al., 2007). In most of the brain disorders described above, Mmps degrade their substrates, such as extracellular matrix (ECM), which then leads to BBB breakdown. Furthermore, Mmp involvement in BBB breakdown is altered in disease states. For example, Mmp9 is elevated in pericytes and endothelial cells in APOE4 more than APOE3 carriers in Alzheimer disease (Halliday et al., 2016). Also, Mmp2 activation has been described as being involved in BBB disruption in ischemia or hypoxia and hemorrhage (Rosenberg, 2012). Thus Mmps and their proteolytic degradation of ECM have been recognized as major risk factors for the breakdown of BBB integrity.

**Figure 1:**
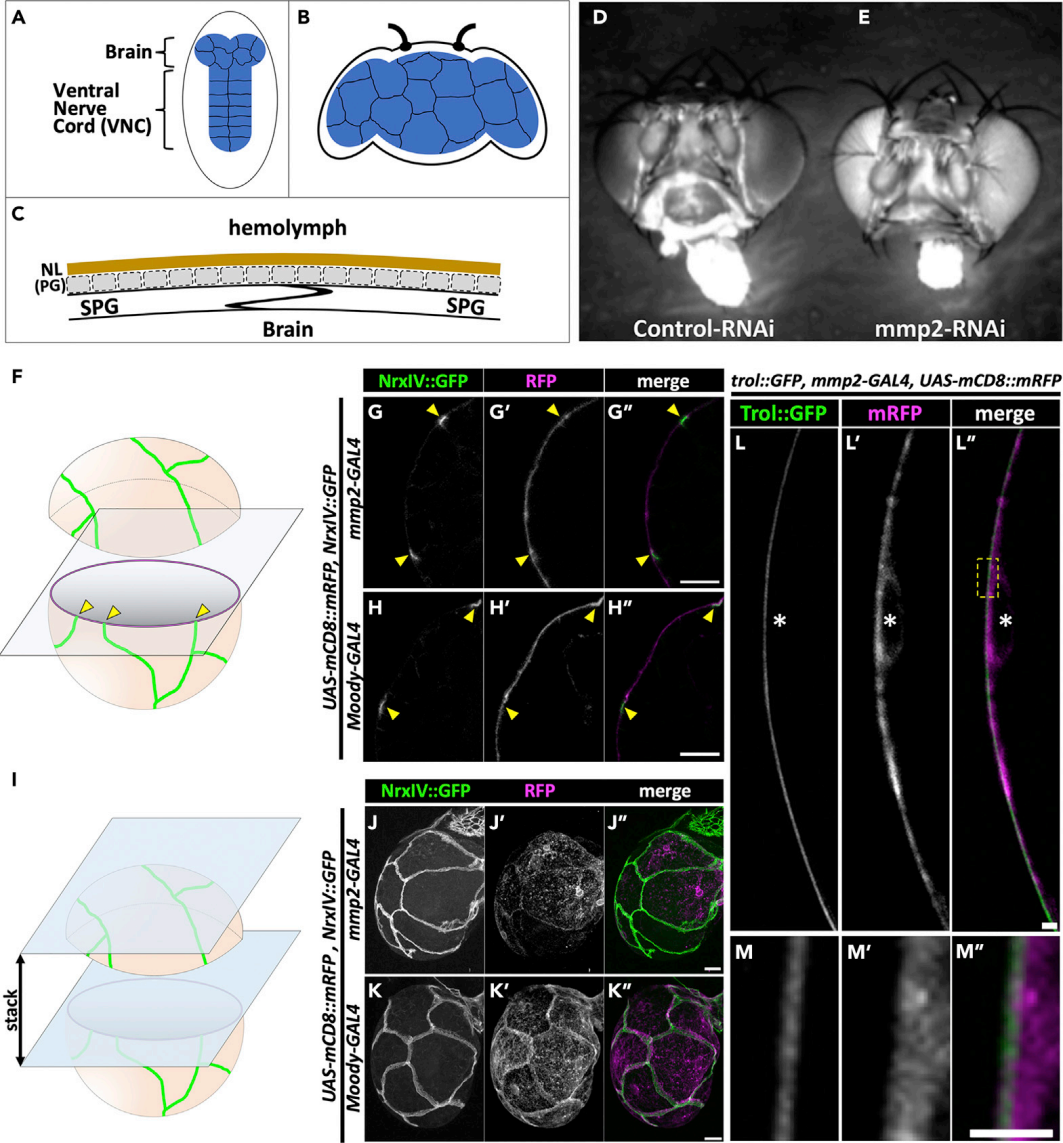
Mmp2 Is Required for the Integrity of the Paracellular Diffusion Barrier (A–C) Schematic representation of the *Drosophila* BBB. BBB-forming subperineurial glia (SPG) ensheathes the brain and ventral nerve cord, a part of the *Drosophila* CNS, by 19–20 h after egg laying (AEL) (Schwabe et al., 2005) (A). SPG are highlighted in blue in (A) and (B). (B) The adult brain and SPG. (C) Schematic lateral view of the *Drosophila* BBB. SPG form septate junctions to prevent paracellular diffusion. PG largely arise post embryonically and divide and cover the surface of the CNS by the late larval to midpupal stages (Awasaki et al., 2008, Stork et al., 2008). (D and E) Leakiness of BBB in SPG-specific *mmp2*-RNAi animals. Two independent dsRNA lines for *mmp2* (NIG 1794-1R-1 and BL61309) showed the same phenotype (E). (F–K″) Mmp2 is expressed in the SPG of the *Drosophila* CNS. (F) Schematic representation of the cross section of the larval brain. Green signal represents the SJ belts. SPG membrane is colored with light orange, and its cross section is colored with magenta. Yellow arrowheads indicate the position where green and magenta signals are colocalized. (G–H″) Cross sections of 12-h-ALH larval brains in which membrane-targeted mRFP was ectopically expressed under the control of *mmp2-GAL4* (G–G″) or *Moody-GAL4* (H–H″) drivers. Note that NrxIV::GFP-positive signal is always colocalized with mRFP signal in both samples. (I) Schematic representation of the image processing that is shown in (J–K″). (J–K″) Stacked images of multiple brain sections at 12 h ALH in which membrane-targeted mRFP was ectopically expressed under the control of *mmp2-GAL4* (J–J″) or *Moody-GAL4* (K–K″) drivers. Note that RFP signal visualizes the lateral membranes of SPG, which is colocalized with the NrxIV::GFP signal in both samples. Expression pattern of endogenous Mmp2 is also shown in Figure S1. (L–M″) Mmp2-expressing cells are juxtaposed to NL. Cross section of 12-h-ALH larval brain whose genotype is *w, trol::GFP/w; mmp2-GAL4/UAS-mCD8::mRFP*. Images of Trol::GFP (L) and mRFP (L′) and merged image (L″). Asterisk indicates the position of nucleus. The higher-magnification images of boxed region in (L″) are shown in (M–M″). Scale bars, 10 μm in (G″, H″, J″, and K″) and 1 μm in (L″ and M″). See also Figures S1 and S9.

Interestingly, even among vertebrates, in some taxa, such as elasmobranchs, the isolating function of the BBB is achieved by glia (Abbott et al., 1986). This “glia-type” BBB is often observed in invertebrates, such as cephalopods and insects (Abbott et al., 1986). In *Drosophila melanogaster*, this function is achieved by a thin layer of subperineurial glia (SPG). SPG surround the CNS and form septate junctions (SJs) by embryonic stage 17 (Schwabe et al., 2005) (Figures 1A–1C) that strictly prevent the paracellular penetration of solutes from circulating hemolymph into the CNS. The outermost surface of the CNS is surrounded by a basement membrane (BM), the neural lamella (NL) (Carlson et al., 2000), which can be detected from embryonic stage 16 onward (Stork et al., 2008). In addition, another cellular layer, which is located between SPG and NL, is achieved by perineurial glia (PG). PG are not thought to contribute to the BBB properties early in development (Hindle and Bainton, 2014) as they largely arise post-embryonically and divide extensively during larval development to cover the surface of the CNS by the late larval to midpupal stages (Awasaki et al., 2008, Stork et al., 2008).

The *Drosophila* genome contains at least seven claudin family of proteins (Nelson et al., 2010), which are the main components of TJs in mammals (Haseloff et al., 2015). Three of these proteins, namely, Megatrachea, Sinuous, and Kune-Kune, are reported to be required for BBB integrity in *Drosophila* (Nelson et al., 2010, Stork et al., 2008). SPG also express one or more ATP-binding cassette transporters, which serve as xenobiotic BBB transporters (Hindle and Bainton, 2014). These considerable similarities, together with the nature of *Drosophila* as a sophisticated genetic tool, provide us with an excellent model system for studying the establishment of the highly orchestrated BBB system (Hindle and Bainton, 2014, Schirmeier and Klambt, 2015).

## Results

### BBB-Specific Knockdown of Mmp2 Results in the Disruption of BBB Integrity

To identify genes that are required for the integrity of BBB in *Drosophila*, we have conducted an *in vivo* RNA interference (RNAi)-based screen (details will be published separately). In principle, BBB-specific knockdown of a list of genes was achieved by the GAL4/UAS (Brand and Perrimon, 1993)-based method. BBB-forming SPG-specific Moody-GAL4 driver (Schwabe et al., 2005) and the stock library of the UAS-double-stranded RNAs (dsRNAs) (NIG-FLY stock center) were used. When fluorescently labeled dextran (10 kDa) was injected as a tracer to monitor the integrity of BBB into the abdomen of adult animals that have intact BBB, it should be excluded from the CNS (Figure 1D) (Bainton et al., 2005). In contrast, when the integrity of BBB was reduced by the SPG-specific RNAi of a gene, the tracer should penetrate into the CNS, resulting in the fluorescence signal from the compound eyes (Figure 1E). Among more than 10,000 lines tested, we found that the SPG-specific knockdown of one of two *Drosophila* Mmps, Mmp2, showed the desired phenotype (Figure 1E). Although the pan-glial expression of *mmp2-dsRNA* is reported to induce the embryonic or early larval lethality (Meyer et al., 2014), the SPG-specific expression of *mmp2-dsRNA* driven by the Moody-GAL4 driver (Moody-GAL4/*UAS-mmp2-dsRNA*) did not induce a significant lethality (the *UAS-mmp2-dsRNA* stocks that were used in this study; NIG1794-1R-1, BL61309, and BL31371).

We then examined the expression pattern of *mmp2*. To this end, a GAL4 enhancer trap in the *mmp2* locus (*mmp2-GAL4;* Srivastava et al., 2007, Wang et al., 2010, Yasunaga et al., 2010; Figure S1) was used to induce the ectopic expression of *UAS-mCD8::mRFP* in which mRFP was fused to the mouse CD8 extracellular and transmembrane domains for membrane targeting. Simultaneously, the SJs of SPG were visualized by a protein trap insertion into the *NrxIV* locus (NrxIV::GFP) (Buszczak et al., 2007, Morin et al., 2001). The NrxIV::GFP signal is confined to the SJs of SPG (Stork et al., 2008). When the cross sections of the brains at 12 h after larval hatching (12 h ALH) were analyzed, the NrxIV::GFP signal always colocalized with the mRFP signal (Figures 1F and 1G″). This finding was also the case when the ectopic expression of *UAS-mCD8::mRFP* was driven by the *Moody-GAL4* driver (Figures 1H–1H″). These results indicate that *mmp2-GAL4* drives the ectopic expression of *UAS-mCD8::mRFP* in the same cell type (SPG) as the *Moody-GAL4* driver. In addition, when multiple z-sections were stacked, then *mmp2-GAL4/UAS-mCD8::mRFP* clearly visualized the lateral membrane of SPG as the signal was colocalized with NrxIV::GFP (Figure 1I–1J″). Again, this colocalization was also observed when the *Moody-GAL4* driver was used (Figures 1K–1K″). Taken together, these results indicate that Mmp2 is expressed in SPG.

We also found that the *mmp2-GAL4/UAS-mCD8::mRFP* signal was surrounded by a dense network of ECM, NL (Carlson et al., 2000) at 12 h ALH, which was visualized by a protein trap insertion into the locus of *Drosophila* heparan sulfate proteoglycan, *trol* (Trol::GFP) (Morin et al., 2001) (Figures 1L–1M″). As most PG arise post-embryonically, and surround the SPG layer below the NL by the late larval to midpupal stages (Awasaki et al., 2008, Stork et al., 2008), it is likely that a substantial region of the SPG membrane could be directly surrounded by NL at this developmental stage.

Together, these results suggested the possibility of a role for Mmp2 in the regulation of BBB integrity, presumably by regulating the mobility of the surrounding NL.

### Mmp2 Is Required for the Establishment of the BBB

**Figure 2:**
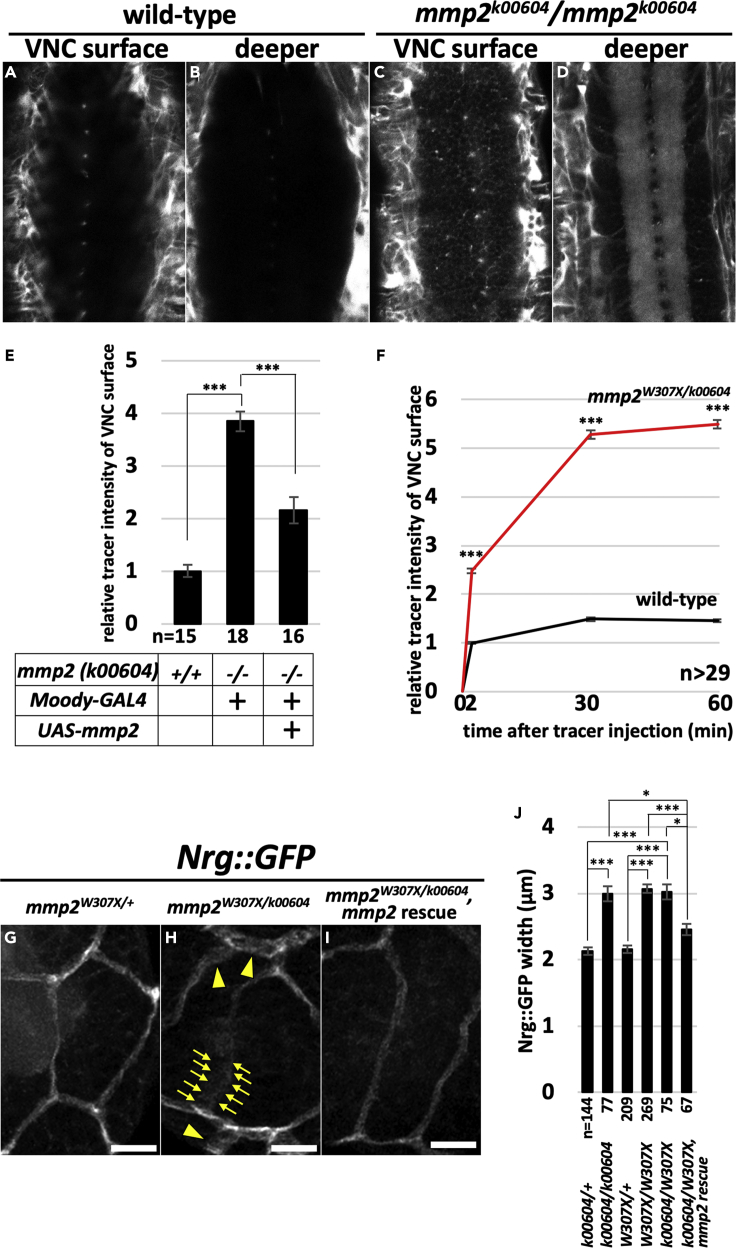
Mmp2 Is Required for Establishment of the BBB (A–D) Texas Red-dextran (10 kDa) did not penetrate into the CNS of stage 17 wild-type embryos (A and B), whereas *mmp2* mutant embryos showed the tracer penetration phenotype (C and D). Images of the ventral surfaces (A and C) and deeper layers (B and D) of the VNCs of the same embryos. Note that tracer penetration was detected in (C), and thus the neuropile in the VNC was stained in the *mmp2* mutant embryo (D). (E) The fluorescence intensity of Texas Red at the ventral surface of the VNC of the indicated genotypes was measured 30 min after tracer injection, and the result was analyzed statistically. The ratios of the mean pixel intensity relative to the wild-type control are shown. (F) Time course analysis of the tracer penetration into the CNS. Texas Red-dextran (10 kDa) was injected into the hemocoel of wild-type or *mmp2* mutant stage 17 embryos, and the mean pixel intensity of the VNC surface was analyzed at each time point. The ratios of the fluorescence intensity relative to the wild-type sample at 2 min after injection are shown. (G–J) (G–I) Representative images of the SJs of control (G), *mmp2* mutant (H), and *mmp2* mutant in which *mmp2* was ectopically expressed in SPG (I) 12-h-ALH brains that were visualized with the Nrg::GFP reporter. Note that the width of the signal is increased in the *mmp2* mutant. The mutant phenotype was significantly suppressed when *mmp2* cDNA was ectopically expressed in SPG (I, and rightmost column of J). The results were analyzed statistically and are shown in (J). The allelic combinations of *mmp2* that were analyzed in the assay are shown in (J). Scale bars, 10 μm (G–I). The results are presented as means ± SEMs. ***p < 0.001, *p < 0.05. See also Figures S2, S6, and S7.

To determine at which developmental stage Mmp2 is required for BBB integrity, BBB integrity was examined in loss-of-function mutants for *mmp2*. To this end, the fluorescent tracer was injected into the hemocoel of stage 17 embryos because SPG insulate the CNS by this developmental stage (Schwabe et al., 2005). The tracer was excluded from the CNS of the wild-type control, but penetrated into the CNS of the *mmp2* mutants (Figures 2A–2D), indicating that the BBB was disrupted in the mutant animals. In addition, ectopic expression of *mmp2* cDNA by *Moody-GAL4* driver in the SPG of the *mmp2* mutant animals significantly suppressed tracer penetration into the CNS (Figure 2E). This result indicates that the gene responsible for the mutant phenotype is *mmp2*. In addition, this result also suggests that although most if not all larvae died by 24 h ALH when *mmp2* cDNA was ectopically expressed driven by the *Moody-GAL4* driver, the lethality of the animal and the integrity of the BBB are likely not necessarily correlated. The fluorescence intensity in the ventral surface of the VNC quickly increased in a time-dependent manner, and was nearly saturated by 30–60 min after injection (Figure 2F).

To elucidate the molecular basis of this phenotype, we analyzed the expression pattern of one of the SJ components, Neuroglian (Nrg), which is a faithful marker of SJ formation (Schwabe et al., 2005, Schwabe et al., 2017). As the thick cuticle of the stage 17 embryo limits the histological analysis of CNS, we analyzed the mutant phenotypes at the early larval stage by taking advantage of the fact that *mmp2* mutants essentially survive until the pupal stage (Page-McCaw et al., 2003, Srivastava et al., 2007). When SJs were visualized with a protein trap insertion into the *Nrg* locus (Nrg::GFP) (Morin et al., 2001), a very thin and distinct GFP signal was detectable along the lateral membranes of the SPG in 12-h-ALH control animals (Figures 2G and S2). In sharp contrast, *mmp2* mutants showed significantly broader GFP signal (Figure 2H, arrowheads; 2J; and Figure S2). In addition, the Nrg::GFP signal was often diffuse and irregular in shape in *mmp2* mutant animals (Figure 2H, highlighted by arrows). Again, this phenotype was rescued by the ectopic expression of *mmp2* cDNA, which was driven by *Moody-GAL4* driver (Figures 2I and 2J). Together, these results indicate that Mmp2 is required for the establishment of the functional BBB.

### Proteolytic Degradation of Particular NL Components Is Critical for the Establishment of the BBB

**Figure 3:**
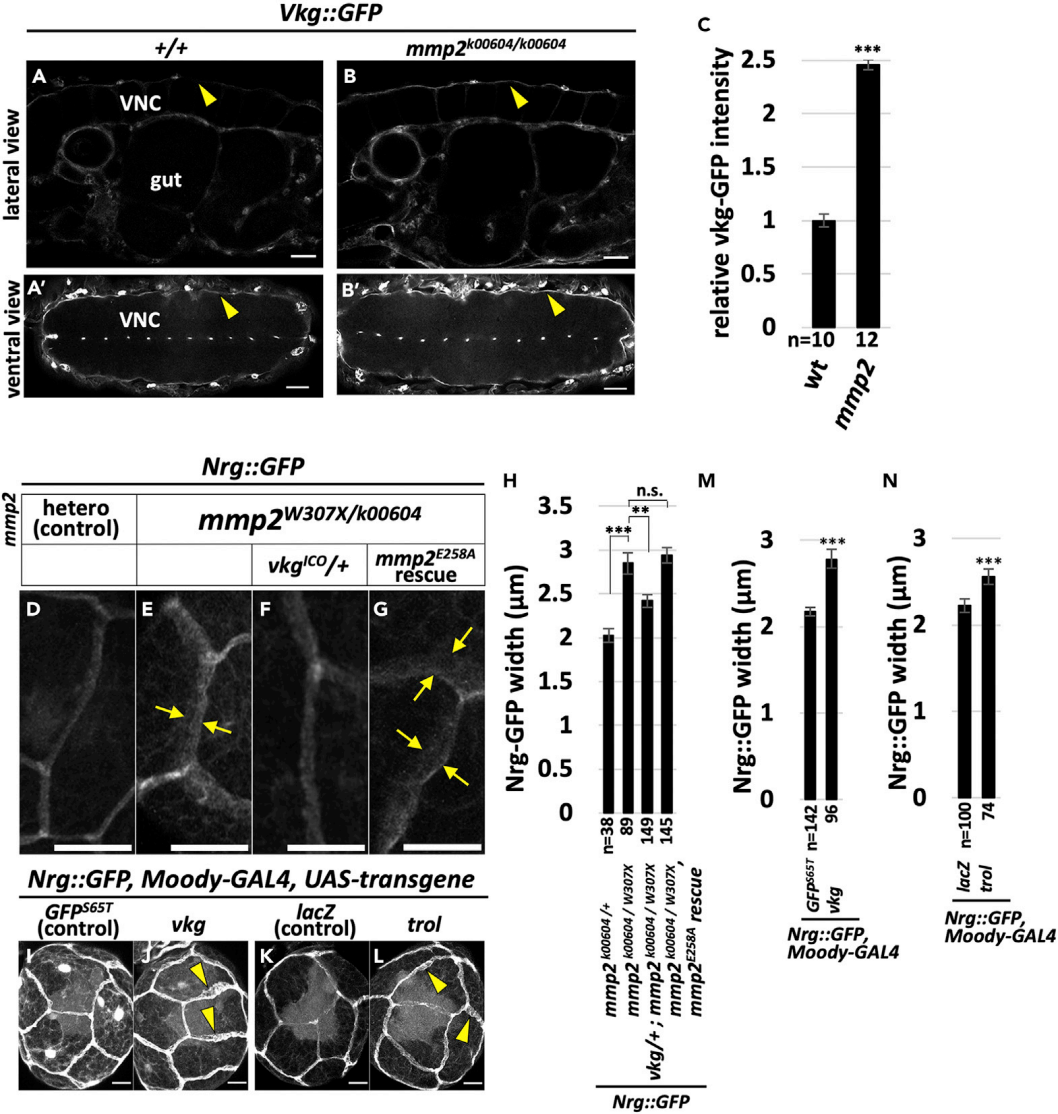
Proteolytic Degradation of Particular NL Components Is Critical for BBB Establishment (A–C) (A and B) The Vkg::GFP patterns of wild-type (A and A′) and *mmp*2 mutant (B and B′) stage 16 embryos. Arrowheads indicate representative regions. Lateral (A and B) and ventral views (A′ and B′) are shown. Ventral is to the top in (A) and (B), and anterior is to the left in (A–B′). The fluorescence intensity of the ventral surface of the VNCs was quantified, and the ratio of the fluorescence intensity relative to the wild-type control is shown in (C). (D–H) Representative images of the SJs of control (D), *mmp2* mutant (E), *mmp2* mutant in the heterozygous mutant background for *vkg*^*ICO*^ (F), and *mmp2* mutant in which *mmp2*^*E258A*^ was ectopically expressed in SPG (G) 12-h-ALH brains that were visualized with the Nrg::GFP reporter. In (E and G) boundaries of diffused signals are highlighted by two arrows. The width of Nrg::GFP in (D–G) was measured and analyzed statistically in (H). (I–N) (I–L) The Nrg::GFP pattern in animals in which each NL component was ectopically expressed in SPG. Data from (I) to (L) were statistically analyzed and are shown in (M) and (N). As the expression vector used in (J) also contains *UAS-GFP*^*S65T*^, *UAS-GFP*^*S65T*^ was used as the control (I). Scale bars, 20 μm in (A–B′) and 10 μm in (D–G and I–L). The results are presented as means ± SEMs. ***p < 0.001, **p < 0.01, n.s., not significant. See also Figures S3–S5.

ECM is the best characterized substrate for the proteolytic activity of Mmps. SPG are surrounded by the CNS BM, NL, when the BBB is established (Schwabe et al., 2005, Stork et al., 2008). We thus aimed to determine whether NL is the functional substrate for Mmp2 in the establishment of the BBB. The four major components of BM/NL are collagen IV, heparan sulfate proteoglycan, Laminin, and Nidogen, all conserved in *Drosophila* (Kalluri, 2003, Pastor-Pareja and Xu, 2011). We first examined the expression level of endogenous collagen IV, also known as Viking (Vkg), in the *mmp2* mutant CNS. To this end, we used a protein trap insertion into the *vkg* locus (Vkg::GFP) (Morin et al., 2001). As the result, we found a significant increase in the intensity of Vkg::GFP in the *mmp2* mutant embryos (Figures 3A–3C), indicating that the increased protein stability of Vkg is due to the loss of Mmp2.

Next, we sought to examine whether NL components, including Vkg, are the functional substrates of Mmp2 for the establishment of the BBB. To this end, the genetic interaction between *mmp2* and *vkg* was analyzed. The Nrg::GFP reporter was used to visualize the structure of SJs. We found that the broad signal of Nrg::GFP, which was observed in the 12-h-ALH *mmp2* mutant animals (Figure 3E), was significantly restored in the *vkg* heterozygous mutant background (Figures 3F and 3H).

The results described above suggest that endogenous NL degradation is required to establish the BBB. Because some Mmps regulate cellular dynamics in a proteolytic-activity-independent manner in mammals (Turunen et al., 2017), we sought to determine whether Mmp2 regulates the establishment of the BBB via the proteolytic activity. Although the SPG-specific ectopic expression of wild-type *mmp2* cDNA in *mmp2* mutant animals significantly restored the Nrg::GFP pattern at 12 h ALH as shown in Figures 2G–2J, the catalytically inactive form of Mmp2 (Miller et al., 2008) did not suppress the Nrg::GFP pattern in mutant animals (Figures 3G and 3H). These data indicate that the proteolytic degradation of NL by Mmp2 is central to the establishment of the BBB.

We next examined whether excess NL is sufficient to alter the establishment of the BBB. As a result, SPG-specific overexpression of *vkg* resulted in an Nrg::GFP signal with a broad, irregular pattern at 12 h ALH (Figures 3J and 3M), mimicking the phenotype of the *mmp2* mutant animals. This unusual pattern was not observed in the control sample (Figure 3I and 3M). To determine whether the overaccumulation of NL components generally has a negative effect on the BBB, we tested whether the overexpression of individual NL components influences the Nrg::GFP pattern. The change in the Nrg::GFP pattern was also observed when Trol was overexpressed (Figures 3L and 3N). Interestingly, however, neither the overexpression of Laminin A (Lan A) nor that of Laminin B1 (Lan B1) altered the Nrg::GFP pattern (Figure S3), suggesting that specific NL components should be dominantly involved in BBB regulation. Together, these results indicate that the regulation of the amount of a particular subset of NL components is critical for BBB establishment.

If the regulation of the amount of NL by Mmp2 is required to modify the endogenous function of NL components in the regulation of BBB establishment, endogenous NL components should also be involved in the regulation of BBB establishment. To examine this possibility, we tested the BBB integrity in the mutants in which each NL component was depleted. Interestingly, a loss-of-function mutant for *vkg* did not show defects in BBB integrity (Figure S4). This was also the case for a loss-of-function mutant for *trol* (Figure S4). These results suggest that Vkg and Trol are dispensable for BBB establishment.

### Mmp1 Does Not Play a Dominant Role in BBB Regulation

The results described above suggested that component-specific alterations in NL by Mmp2 are critical for the regulation of BBB integrity. Because the *Drosophila* genome encodes two Mmp family of proteins, namely, *mmp1* and *mmp2* (Page-McCaw et al., 2003), we also analyzed the BBB integrity in *mmp1* mutant animals. *mmp1* mutant embryos did not show tracer penetration into the CNS (Figure S5). In addition, we observed only a subtle change in the width of Nrg::GFP signal in the 12-h-ALH *mmp1* mutant brains (Figure S5). Together, these results suggest that Mmp1 does not mirror the function of Mmp2 in BBB establishment.

### Local Mmp2 Activity Regulates the BBB

As shown in Figure 1, SPG-specific knockdown of *mmp2* resulted in a decrease in BBB integrity. To determine the molecular basis of this decrease, we examined whether the BBB structure is impaired when *mmp2* expression in SPG is specifically downregulated. SPG-specific overexpression of dsRNAs for *mmp2* induced a phenotype that was similar to that of the *mmp2* mutant animals (Figures S6A–S6D and S6G). In addition, SPG-specific overexpression of the tissue inhibitor of metalloproteases (TIMP), which inhibits the activity of both Mmp1 and Mmp2 (Wei et al., 2003), resulted in abnormalities in the Nrg::GFP signal that were indistinguishable from those in the *mmp2*-RNAi animals (Figures S6E, S6F, and S6H). These results indicate that local Mmp2 activity regulates BBB establishment.

### Mmp2 Mutation Impairs the Process of Mesenchymal-Epithelial Transition of SPG

**Figure 4:**
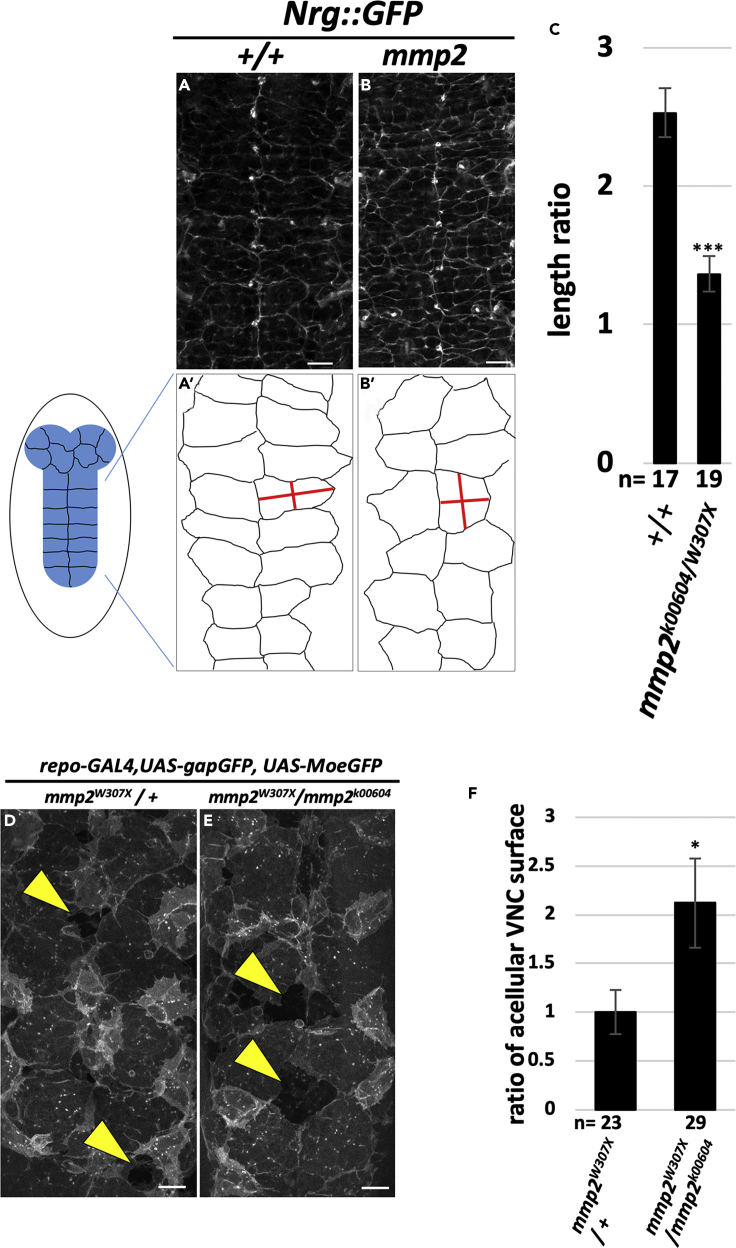
The *mmp2* Mutant Shows a Defect in the Mesenchymal-Epithelial Transition Process of SPG (A–C) Nrg::GFP images of 18–19 h AEL wild-type (A) and *mmp2* mutant (B) embryos. The SPG shape around the VNCs was traced and is shown in (A′) and (B′). The ratio of the medial-lateral to anterior-posterior length of SPG was quantified and is shown in (C). (D–F) The *mmp2* mutants showed a significantly larger acellular VNC surface. To detect very thin SPG layer, the SPG were visualized as described in Schwabe et al. (2017). Representative images of the 13–14 h AEL embryos of heterozygous control (D) and *mmp2* mutant (E) are shown. (F) The area of the acellular regions of the VNC surface (arrowheads) in (D) and (E) was measured and statistically analyzed. The ratio of acellular region compared with the control is shown. Scale bars, 10 μm (A, B, D, and E). The results are presented as means ± SEMs. ***p < 0.001, *p < 0.05. See also Figure S8.

The above results indicate that BBB integrity is not established in the absence of *mmp2*. We sought to determine the step(s) at which Mmp2 activity is required in this process. We first analyzed the morphology of SPG. Careful examination of 18- to 19-h-after-egg-laying (AEL) embryos, in which SJs were visualized with the Nrg::GFP reporter, revealed that SPG in the control VNC were columnar in shape and aligned regularly (Figures 4A and 4A′). In contrast, SPG in the *mmp2* mutant animals often showed irregularities in shape at the same time point (Figures 4B, 4B′, and 4C). Recently, Schwabe et al. (2017) revealed that mutations in genes that are involved in G-protein-coupled receptor signaling result in defects in the process of mesenchymal-epithelial transition (MET) of SPG. We thus examined whether the MET of SPG is altered in the absence of *mmp2*. The MET process occurs from about 9 to 19 h AEL at 25°C. In brief, SPG migrate to the CNS surface (9–11 h), become stationary and cover most of the CNS and begin to contact their neighbors (by 13 h), and largely complete the epithelial closure (14.5–15.5 h) (Schwabe et al., 2017). In the *mmp2* heterozygous control, VNC was almost completely covered by SPG together with PG by 13–14 h AEL (Figure 4D). This was consistent with the findings of Schwabe et al. (2017). In contrast, we detected a significantly larger acellular VNC surface in *mmp2* mutant embryos at the same time point (Figures 4E and 4F), indicating the incomplete MET of SPG in *mmp2* mutants.

## Discussion

In this study, we show that Mmp2 is essential for the establishment of a tightly ensheathed BBB *in Drosophila*.

Regulation of the amount of particular NL components by Mmp2, which is shown in Figures 3 and S3, may lead to the regulation of one or more specific intracellular downstream targets that are required for BBB integrity. Neurexin IV (NrxIV) is another SJ component that is required for BBB integrity (Buszczak et al., 2007, Nilton et al., 2010). Moreover, NrxIV forms a protein complex with Nrg in the nervous system (Banerjee et al., 2006). We thus examined whether Mmp2 also regulates the localization of NrxIV in SJs. Interestingly, however, we found that the expression pattern of endogenous NrxIV was not altered in the 12-h-ALH *mmp2* mutant brains (Figure S7). Although the expression level and localization of core components of the SJ are thought to have an interdependent relationship (Izumi and Furuse, 2014), our result suggests that they could be regulated independently in some contexts. This finding is consistent with the recent report of Paul et al. (2003) in which they showed that neither the expression nor the localization of ATPα is altered in the salivary gland of *coracle* mutants. Regarding the integrity of the BBB in brain, Mmp2 may regulate the intracellular signaling pathway that specifically regulates the proper localization of Nrg.

Recently, Babatz et al. reported that the SJ belts seem to be rather “defasciculated” already in the first instar larval brain, and stretches as the cells grow (Babatz et al., 2018). Thus it is possible that the widening phenotype of Nrg::GFP in *mmp2* mutants (Figures 2H and 2J) might be caused by a developmental delay of SPG. To examine this, we checked the width of Nrg::GFP in the 0-h-ALH brains. If the phenotype in *mmp2* mutants was caused by the developmental delay (or if the width of Nrg::GFP narrows as the larvae develop), then the width of Nrg::GFP of the 12-h-ALH brains should be narrower than that of the 0-h-ALH brains in wild-type animals. As a result, the width of Nrg::GFP in wild-type larval brains was not reduced but rather increased as they developed, indicating that the mutant phenotype was not the developmental delay. These findings are not in conflict with the findings by Babatz et al. because since they did not have several time points in L1 stage. In addition, it is also possible that the dynamics of NrxIV, which they focused, and that of Nrg, which we focused, are regulated independently.

An orphan G-protein-coupled receptor, Moody, is one of the most commonly studied molecules that regulates BBB integrity in *Drosophila* (Babatz et al., 2018, Bainton et al., 2005, Schwabe et al., 2005, Schwabe et al., 2017). Recently, Schwabe et al. (2017) revealed that Moody and its downstream signaling molecules are required for the MET process of SPG; *Moody* mutant animals showed a delay in CNS epithelial closure by SPG. This phenotype is similar to those we found in the *mmp2* mutant animals. Despite the phenotypic similarity between the *mmp2* and *Moody* mutants, we did not find a significant defect in the acquisition of apicobasal polarity during the MET of *mmp2* mutant SPG (Figure S8). This lack of defect suggests that Mmp2 does not regulate the same intracellular signaling pathway(s) as Moody. This difference may be attributable to the different subcellular localizations of Moody and Mmp2; Moody specifically localizes to the brain-facing membrane of the SPG (Mayer et al., 2009, Schwabe et al., 2017), whereas our genetic analysis shows that Mmp2 works at least on the hemolymph-facing membrane of the SPG because Mmp2 and NL components show multiple interactions (Figure 3). In addition, Schwabe et al. (2017) described the importance of synchronized growth behavior of SPG for generating an evenly sealed BBB (Schwabe et al., 2017). Thus it is possible that the widening phenotype of Nrg::GFP may be correlated to the defect of MET in the *mmp2* mutants.

The overexpression of *UAS-mmp2*^*E258A*^ by *Moody-GAL4* driver, which was used in the experiment of Figure 3G, induced lethality by 24–48 h ALH. This is essentially consistent with the report of Meyer et al. (2014). However, in a series of experiments, we noticed that the lethality and the integrity of the BBB are not necessarily correlated. For example, although the null allele for *vkg* (*vkg*^*ICO*^) shows embryonic lethality, their BBB was “intact” at the late embryonic stage, as shown in Figure S4. This result suggests that these mutant animals are dying with an “intact” BBB. When *UAS-mmp2*^*E258A*^*/UAS-mmp2* ^*E258A*^ line was crossed to *Moody-GAL4/CyO,mCherry* line, 12.5% of the offspring were the animals whose genotype was *Moody-GAL4/UAS-mmp2* ^*E258A*^ at 24 h ALH (8 larvae of 64). When *UAS-mmp2/UAS-mmp2* was examined in the same assay, only 3.8% (4 larvae of 104) was animals with expected genotype. Thus although *UAS-mmp2*^*E258A*^ was unable to rescue the Nrg::GFP phenotype of the *mmp2* mutant (Figure 3G), this failure should not be due to the animal lethality because the overexpression of “more toxic” wild-type Mmp2 was able to rescue it (Figures 2I and 2J).

We also found that the expression level of *mmp2*, which was examined by *mmp2-GAL4/UAS-mCD8::mRFP*, was increased in the post-embryonic stages (Figure S9), suggesting that an age-dependent change in the regulatory mechanism of the integrity of the BBB might occur.

Although the Mmp family of proteins has primarily been recognized as a group of major risk factors for the disruption of BBB integrity (Rempe et al., 2016), their endogenous significance in BBB regulation has not been extensively documented in mammals. One possible interpretation for this missing link may be that the mammalian genome contains at least 23 Mmp family members (Page-McCaw et al., 2007), which may make their functions more redundant. In addition, some mammalian Mmps are expressed in vascular endothelial cells (Chen et al., 2013), suggesting the hypothetical significance of Mmps in the regulation of the integrity of the BBB. However, because some Mmps are also required for angiogenesis, mostly if not always in postnatal vascular remodeling and angiogenesis (Page-McCaw et al., 2007, Rodriguez et al., 2010, Rundhaug, 2005), loss of angiogenesis in these *mmp* mutants would hinder the characterization of subsequent events, including the establishment of the BBB. Thus our results, together with the comparative simplicity of the two-member family of Mmp proteins in *Drosophila*, may provide the impetus for more comprehensive studies of previously undiscovered aspects of this protein family in the context of BBB regulation. This genetic approach should further provide clues for testing the functional significance of the Mmp family of proteins in the development of the mammalian BBB.

### Limitations of the Study

In our study, we demonstrated that Mmp2 is required for the establishment of the BBB in *Drosophila*. However, the intracellular signaling molecules that work downstream of Mmp2 remain to be determined. The very thin SPG (<1 μm), and therefore the very small amount of cytosolic proteins in SPG, might cause the technical limitation to detect the slight change in the expression level of intracellular molecules.

## Methods

All methods can be found in the accompanying Transparent Methods supplemental file.

## Supplemental Information

Document S1. Transparent Methods and Figures S1–S9
