# Supplementary material for: Degradation of Extracellular Matrix by Matrix Metalloproteinase 2 Is Essential for the Establishment of the Blood-Brain Barrier in Drosophila
Source: iScience. 2019 May 27;16:218–29. doi: 10.1016/j.isci.2019.05.027 (PMC6562144; doi:10.1016/j.isci.2019.05.027)
Supplement: Document S1. Transparent Methods and Figures S1–S9 [file mmc1.pdf]

ISCI, Volume *16*

Supplemental Information

Degradation of Extracellular Matrix by Matrix Metalloproteinase 2 Is Essential for the Establishment of the Blood-Brain Barrier in *Drosophila*

Hiroshi Kanda, Rieko Shimamura, Michiko Koizumi-Kitajima, and Hideyuki Okano

Supplemental Figures

Figure S1

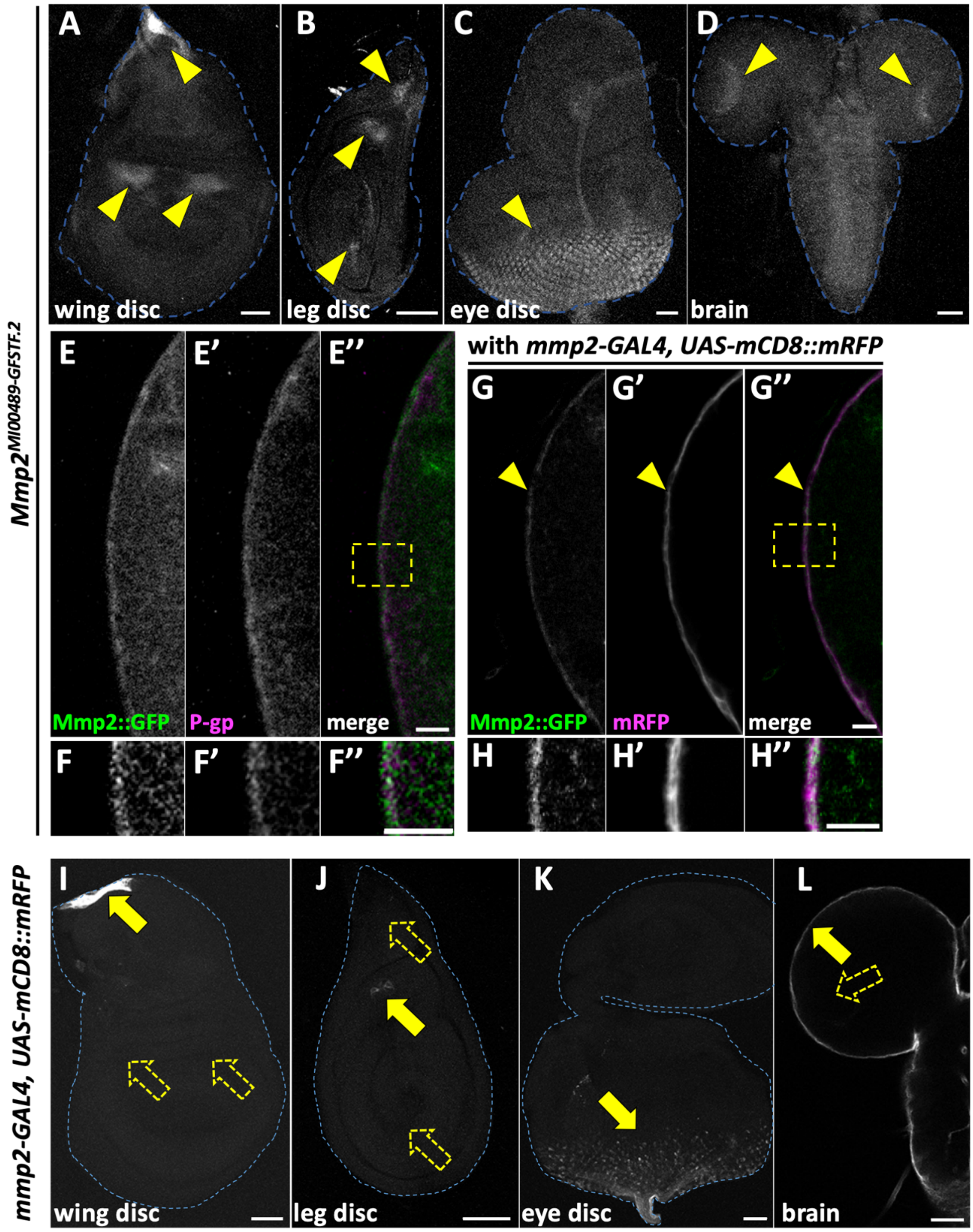

Figure S1. Mmp2 is expressed in SPG, related to Figure 1.

(A-D) Expression pattern of endogenous Mmp2::GFP in *Mmp2*$^{MI00489\text{-}GFSTF.2}$. Representative images of wing disc (A), leg disc (B), eye disc (C), and central nervous system (D) of 3rd instar larvae are shown. Mmp2::GFP signals are indicated by arrowheads. (E-F”) anti-P-glycoprotein (P-gp) immunostaining of the 3rd instar larval brain of *Mmp2*$^{MI00489\text{-}GFSTF.2}$. Boxed region in (E”) is shown in (F-F”). Note that the signals of Mmp2::GFP and P-gp are colocalized in (E” and F”). (G-L) Expression pattern of *mmp2-GAL4*/*UAS-mCD8::mRFP*. mRFP-positive regions that are indicated by solid arrows are also positive for the Mmp2::GFP in *Mmp2*$^{MI00489\text{-}GFSTF.2}$ (A-D, G”). Empty arrows indicate the region where endogenous Mmp2 is supposed to be expressed. Note that the Mmp2::GFP-positive regions are not always mRFP-positive (empty arrows in I, J, and L), however, the mRFP-positive regions are always GFP-positive, suggesting that *mmp2-GAL4* should be the enhancer trap line of one or more, but not all, of *mmp2* isoforms. (G-H”) *mmp2-GAL4*/*UAS-mCD8::mRFP* signal is colocalized with the Mmp2::GFP signal. Boxed region in G” is shown in (H-H”). Scale bars represent 50μm (A-D and I-L), and 10μm (E-H”).

Figure S2

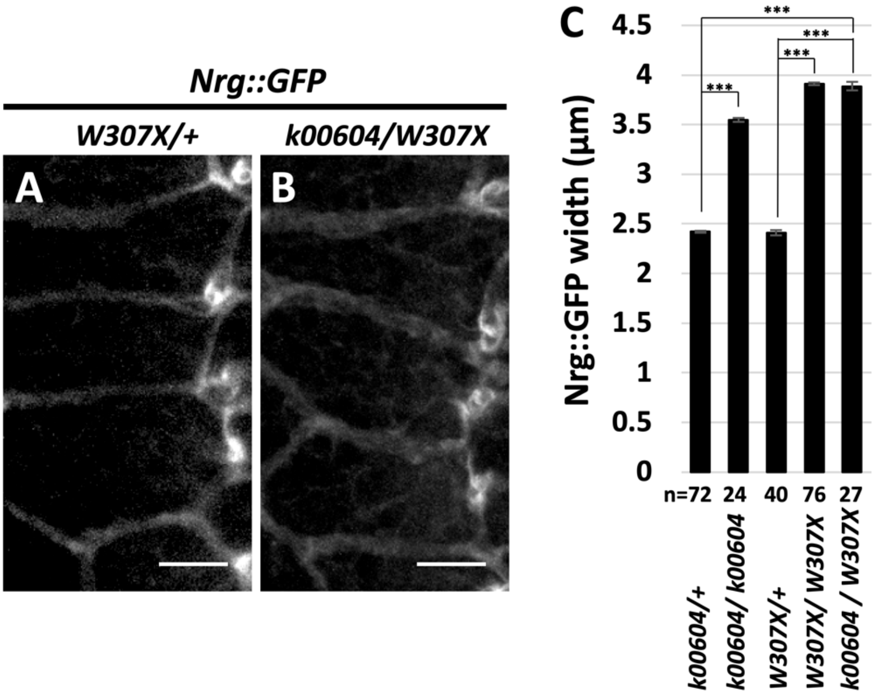

Figure S2. Nrg::GFP is also widened in the VNCs, related to Figure 2.

The width of Nrg::GFP signal was increased in the *mmp2* mutant VNC (B) compared to the heterozygous control (A). The width of Nrg::GFP signal in (A) and (B) was measured and statistically analyzed (C). The combination of mutant alleles that were examined are shown in (C). The results are presented as the means±SEMs. ***p<0.001, Scale bars represent 10 μm.

Figure S3

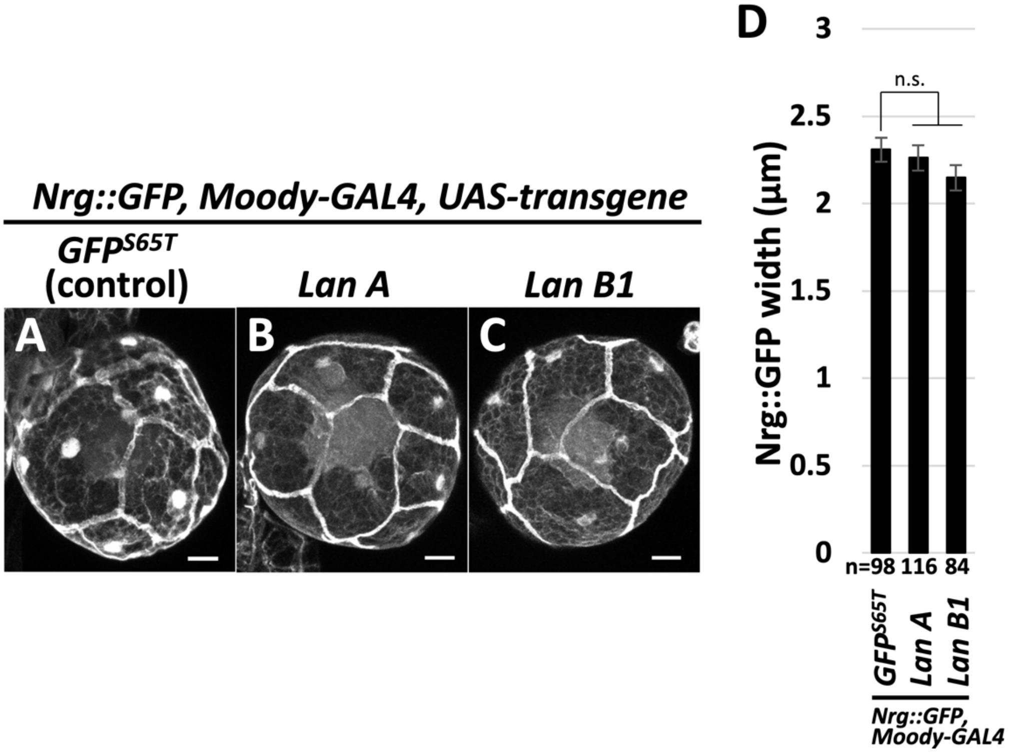

Figure S3. Neither the ectopic expression of Lan A nor that of Lan B1 alters the width of Nrg::GFP, related to Figure 3.

The indicated transgenes were ectopically expressed driven by *Moody-GAL4* driver. Because the expression vectors used in (B) and (C) (GSV6) also contain *UAS-GFP*^{S65T}, *UAS-GFP*^{S65T} was used as the control (A). Scale bars represent 10 μm. The width of Nrg::GFP signal in (A-C) was measured and statistically analyzed (D). The results are presented as the means±SEMs. n.s., not significant.

Figure S4

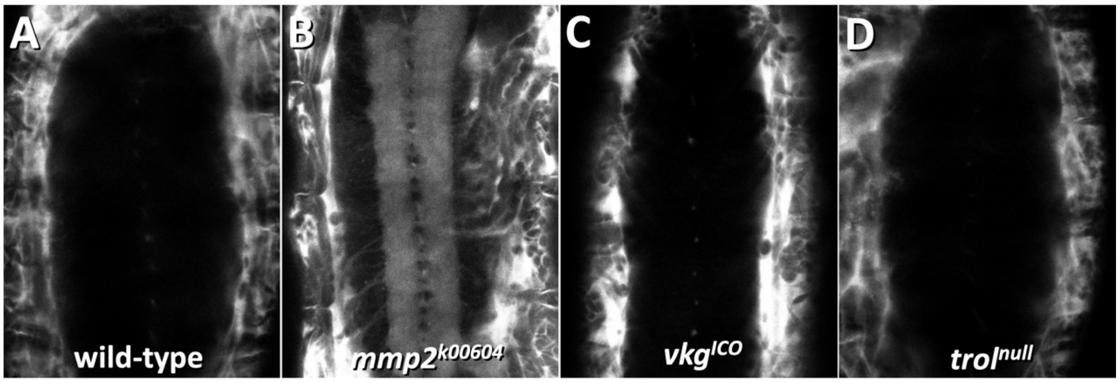

Figure S4. Loss-of-function mutants for *vkg* and *trol* do not show the leaky BBB phenotype, related to Figure 3.

Fluorescence images of stage 17 embryos of wild-type (A), *mmp2*^{k00604} (positive control) (B), *vkg*^{ICO} (C), and *trol*^{null} (D) mutants whose hemocoel was injected with Texas Red-Dextran (10 kDa). Images were obtained 30 minutes after injection.

Figure S5

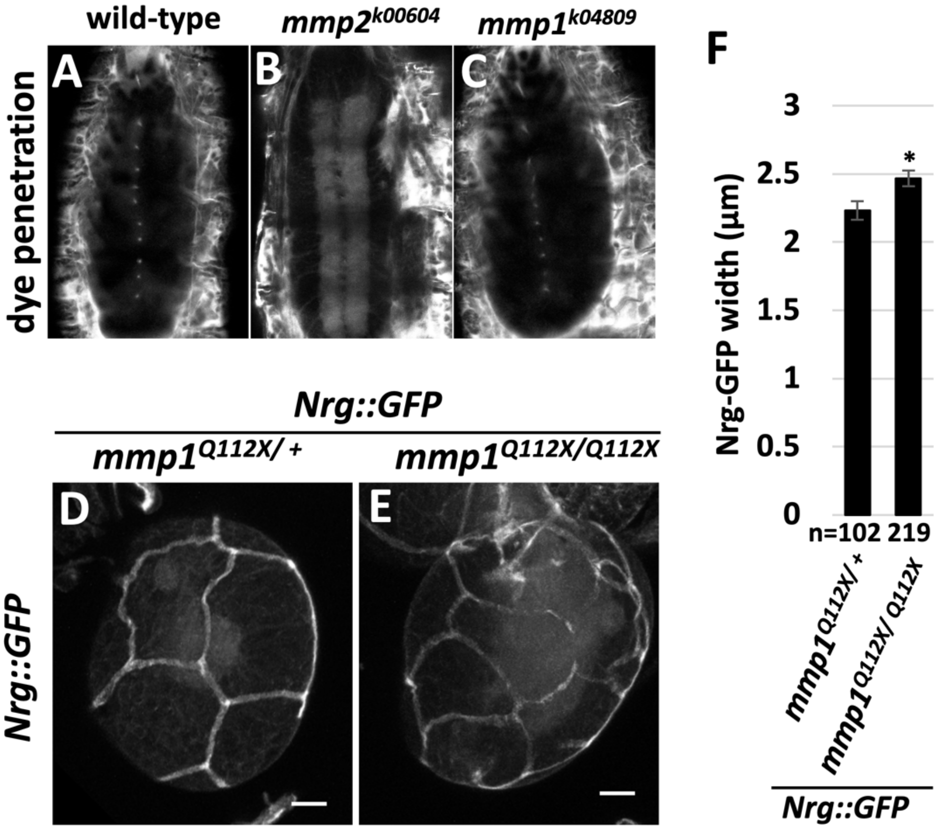

Figure S5. Mmp1 is not predominantly involved in the establishment of BBB integrity, related to Figure 3.

(A-C) Texas Red-Dextran (10 kDa) was excluded from the CNS of the wild-type (A) and *mmp1* mutant stage 17 embryos (C) but not from that of the *mmp2* mutant embryos (positive control) (B). (D and E) The Nrg::GFP pattern in heterozygous control (D) and *mmp1* mutant 12-hr-ALH brains (E). The width of Nrg::GFP signal was measured and statistically analyzed (F). Scale bars represent 10 µm. The results are presented as the means±SEMs. *$p<0.05$.

Figure S6

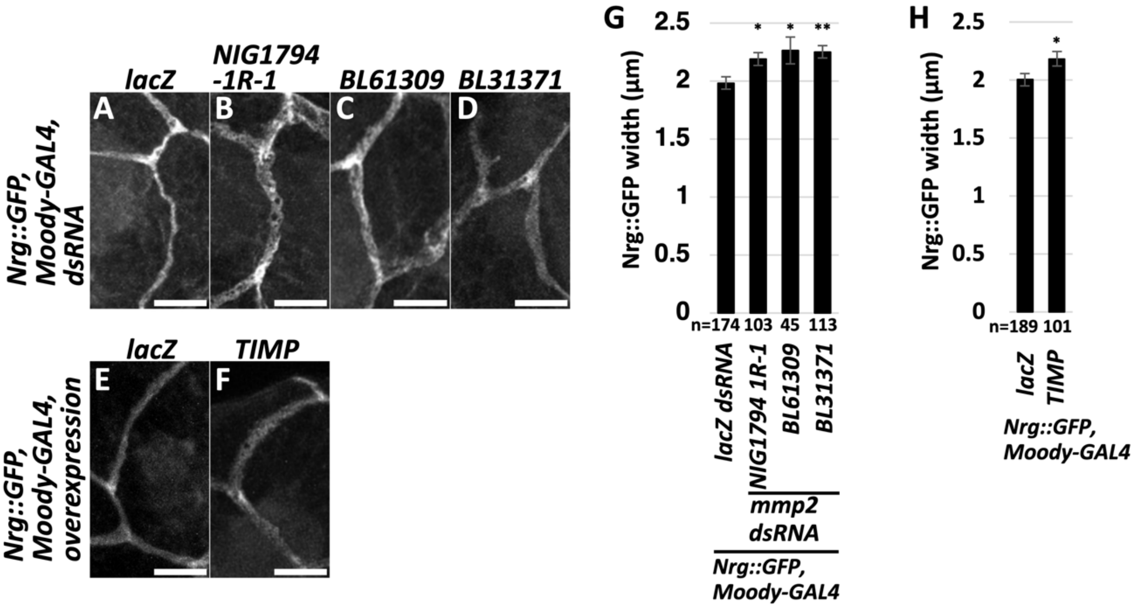

Figure S6 Local Mmp2 is required for the establishment of proper SJs, related to Figure 2. Representative images of the SJs of control (A and E) and SPG-specific *mmp2*-RNAi (B-D) or overexpression of TIMP (F) that were visualized by the Nrg::GFP reporter. The 12-hr-ALH brains were analyzed. The stock numbers of transgenic lines that were used to induce RNAi were NIG1794-1R-1 (B), BL61309 (C), and BL31371 (D). The width of the Nrg::GFP signal was measured and statistically analyzed (G and H). Scale bars represent 10 μm. The results are presented as the means±SEMs. $^{**}p<0.01$, $^{*}p<0.05$.

Figure S7

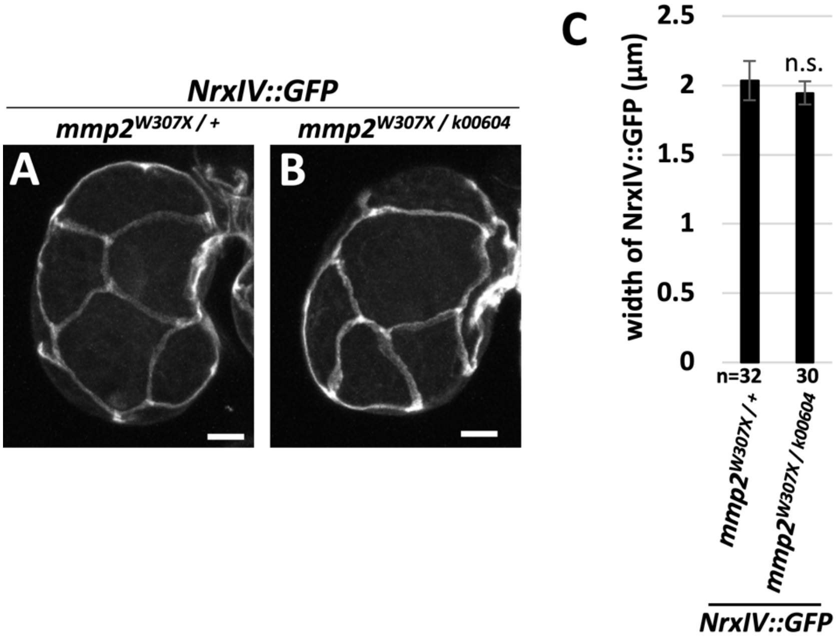

Figure S7. ***mmp2*** **mutant brains do not show a defective pattern in NrxIV::GFP, related to Figure 2.**

The NrxIV::GFP patterns of the *mmp2* heterozygous control (A) and *mmp2* mutant (B) are shown. The width of the Nrg::GFP signal was measured and statistically analyzed (C). Scale bars represent 10 µm. The results are presented as the means±SEMs. n.s., not significant.

Figure S8

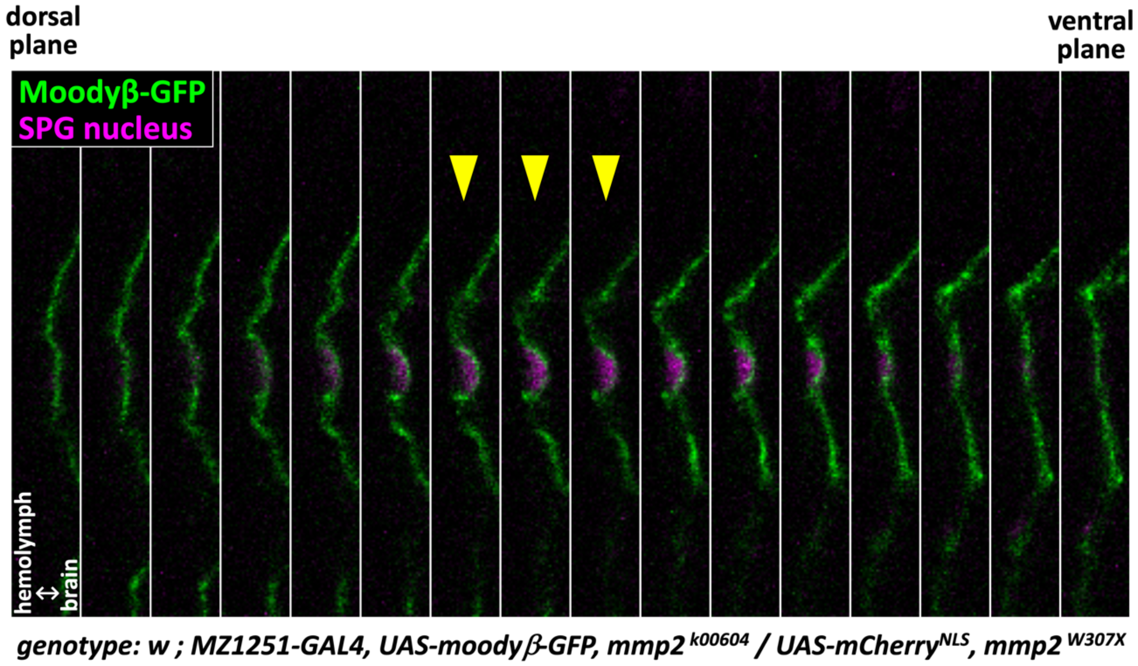

Figure S8. The *mmp2* mutation does not affect the acquisition of the apicobasal polarity of SPG, related to Figure 4.

Serial sections of the indicated genotype are shown. The left side is the hemolymph-facing side of the SPG in each panel, and the left-most panel is the image of the dorsal-most section of the brain hemisphere of the stage17 *mmp2* mutant embryo. In wild-type embryos, the SPG acquire apicobasal polarity in terms of the specific distribution of Moody in the brain-facing side of the SPG membrane by embryonic stage 17 (Schwabe et al., 2017). If the SPG do not acquire apicobasal polarity in *mmp2* mutants, then the Moodyβ-GFP signal should be detected at both brain-facing and hemolymph-facing sides of the membranes, which makes the nucleus 'sandwiched' by the Moodyβ-GFP signals. This phenotype should be most clearly detected in panels where both sides of the plasma membranes are on the equatorial plane of the nucleus. These panels are indicated by the arrowheads. We found that the Moodyβ-GFP signal was observed only at the brain-facing side of the nuclei, indicating that the SPG acquired apicobasal polarity in the *mmp2* mutants.

Figure S9

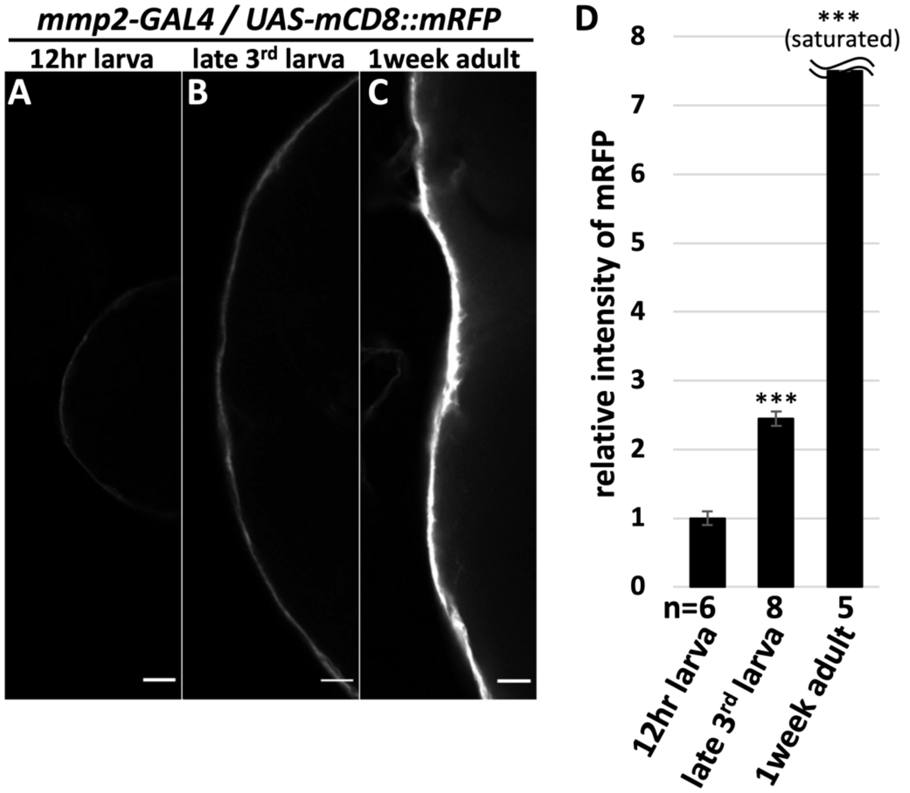

Figure S9. Change in the expression level of *mmp2*, related to Figure 1. Representative images of the cross sections of 12-hr-ALH larval (A), late 3rd instar larval (B), and 1 week old adult (C) brains of the *mmp2-GAL4/UAS-mCD8::mRFP* are shown. Images are taken with the same detection condition. The fluorescence intensity was measured, and analyzed statistically (D). The ratio of the fluorescence intensity relative to the 12 hr larval sample are shown. The results are presented as the means±SEMs. ***p<0.001, Scale bars represent 10μm.

Transparent Methods

Fly genetics

Fly cultures and genetic analysis were performed at 25°C unless otherwise specified. w^{1118} or mutant heterozygotes were used as the control.

Histology

Samples were dissected in PBS and fixed in 4% paraformaldehyde-PBS. Washes were performed with 0.5% Triton X-100-PBS. Fixed samples were stained according to the standard protocol using anti-P-glycoprotein antibody (mouse monoclonal, clone C494, 1:200, ThermoFisher SCIENTIFIC). The secondary antibody was Alexa555 anti-mouse IgG (1:1,000, Invitrogen). Images were obtained by confocal laser microscopy (LSM700, Zeiss). For the quantification of the width of Nrg::GFP and NrxIX::GFP, the GFP signal of each serial section of brain hemispheres was carefully traced using Zen software (Zeiss), and the widest part of the signal of each SJ belt of SPG was measured. Multiple SJ belts from at least six brains were examined and statistically analyzed to exclude any bias. The quantification of the Nrg::GFP signal of the VNC followed the same method. For the quantification of the intensity of Vkg::GFP, the GFP signal of a representative plane of each sample was acquired by confocal laser microscopy (LSM 700, Zeiss). The intensity of the signal was quantified on the ZEN software (Zeiss), and statistically analyzed relative to the intensity of the wild-type control. For the quantification of the ratio of medial-lateral to anterior-posterior length of SPG, at least four SPG from three independent embryos were examined in each sample.

Dye penetration assay

For adults, Texas Red-conjugated Dextran (10 kDa, Invitrogen) was injected into the abdomens of adult animals according to the method of (Bainton et al., 2005). Images of adult eyes were obtained 24 hrs after injection. For embryos, Texas Red-conjugated Dextran (10 kDa, Invitrogen) was injected into the hemocoel of 19-20 hr (stage 17) embryos, and images were obtained 30 minutes after injection by confocal laser microscopy (LSM700, Zeiss). For the time-course assay in Figure 2F, images were obtained at the indicated time points. The fluorescence intensity of the ventral surface of the VNCs was measured using photoshop software.

Quantification of the MET

The images were obtained using confocal laser microscopy (LSM700, Zeiss). To increase the signal intensity, the pinhole was opened to 1.3 according to (Schwabe et al., 2017). Thirteen to seventeen sections were acquired per sample, and the acellular regions on the stacked images were quantified with ImageJ (NIH).

Statistical analysis

Statistical analysis was performed with SPSS Statistics (IBM). The following programs were used: one-way ANOVA with a post hoc Tukey test for Figure 2E, 2J, 3H, and S2C; one-way ANOVA with a post hoc Dunnett's test for Figure S3D, S6G and S9D; and the unpaired Student's *t*-test for Figure 2F, 3C, 3M, 3N, 4C, 4F, S5F, S6H, and S7C. Significance was accepted at $p<0.05$.

Drosophila **stocks**

w^{1118}	Bloomington Drosophila Stock Center (BDSC)	5905
Moody-GAL4	University of California, San Francisco	Roland Bainton
repo-GAL4	BDSC	7415
mmp2-GAL4	Drosophila Genomics and Genetic Resources (DGGR, Kyoto Institute of Technology)	103625
MZ1251-GAL4	The University of Tokyo	Kei Ito
UAS-GFP^{S65T}	BDSC	1521
UAS-mCherry^{NLS}	BDSC	38425
UAS-mCD8::mRFP	BDSC	27398
UAS-lacZ	The University of Tokyo	Masayuki Miura
UAS-mmp2	Vanderbilt University	Andrea Page-McCaw
UAS-mmp2^{E258A}	Vanderbilt University	Andrea Page-McCaw
UAS-trol	DGGR	201233
UAS-vkg	DGGR	206046
UAS-LanA	DGGR	207443
UAS-LanB1	DGGR	205294
UAS-gapGFP	BDSC	4522
UAS-MoeGFP	Duke University	Dan Kiehart
UAS-lacZ dsRNA	The University of Tokyo	Masayuki Miura
UAS-mmp2 dsRNA$^{NIG1794\text{-}1R\text{-}1}$	National Institute of Genetics Stock Center (NIG-FLY)	1794-1R-1
UAS-mmp2 dsRNA^{BL31371}	BDSC	31371
UAS-mmp2 dsRNA^{BL61309}	BDSC	61309
UAS-TIMP	Vanderbilt University	Andrea Page-McCaw
UAS-moodyβ-GFP	University of California, San Francisco	Roland Bainton
mmp2^{k00604}	BDSC	10358

mmp2^{W307X}	Vanderbilt University	Andrea Page-McCaw
mmp1^{k04809}	BDSC	12205
mmp1^{Q112X}	Vanderbilt University	Andrea Page-McCaw
vkg^{ICO}	University of California, Davis	Deborah Kimbrell
trol^{null}	Max Planck Institute	Doris Brentrup
Nrg::GFP	BDSC	6844
vkg::GFP	DGGR	110626
NrxIV::GFP	BDSC	50798
trol::GFP	DGGR	110836
Mmp2$^{MI00489\text{-}GFSTF.2}$	BDSC	60512

Sample genotypes

Figure 1	
(D)	w; Moody-GAL4 / UAS-lacZ dsRNA
(E)	w; Moody-GAL4 / + ; UAS-mmp2 dsRNA $^{NIG1794\text{-}1R\text{-}1}$ / +
(G-G” and J-J”)	w; mmp2-GAL4 / UAS-mCD8::mRFP ; NrxIV::GFP / +
(H-H” and K-K”)	w; Moody-GAL4 / UAS-mCD8::mRFP ; NrxIV::GFP / +
(L-M”)	w, trol::GFP / w ; mmp2-GAL4 / UAS-mCD8::mRFP
Figure 2	
(A, B)	w^{1118}
(C, D)	w; mmp2^{k00604} / mmp2^{k00604}
(E, left)	w^{1118}
(E, middle)	w; Moody-GAL4, mmp2^{k00604} / mmp2^{k00604}
(E, right)	w; Moody-GAL4, mmp2^{k00604} / UAS-mmp2, mmp2^{k00604}
(F)	w^{1118} (wild-type) and w; mmp2^{W307X} / mmp2^{k00604}
(G)	w, Nrg::GFP / w ; mmp2^{W307X} / +
(H)	w, Nrg::GFP / w ; mmp2^{W307X} / mmp2^{k00604}
(I)	w, Nrg::GFP / w ; Moody-GAL4, mmp2^{W307X} / UAS-mmp2, mmp2^{k00604}
Figure 3	
(A, A’)	w; vkg::GFP / vkg::GFP
(B, B’)	w; vkg::GFP, mmp2^{k00604} / vkg::GFP, mmp2^{k00604}

(D)	w, Nrg::GFP / w ; mmp2 ^{k00604} / +
(E)	w, Nrg::GFP / w ; mmp2^{W307X} / mmp2^{k00604}
(F)	w, Nrg::GFP / w ; vkg^{ICO}, mmp2 ^{W307X} / mmp2 ^{k00604}
(G)	w, Nrg::GFP / w ; Moody-GAL4, mmp2 ^{W307X} / UAS-mmp2^{E258A}, mmp2 ^{k00604}
(I)	w; Nrg::GFP/ w ; Moody-GAL4 / UAS-GFP^{S65T}
(J)	w; Nrg::GFP/ w ; Moody-GAL4 / UAS-vkg^{DGGR206046}
(K)	w; Nrg::GFP/ w ; Moody-GAL4 / UAS-lacZ
(L)	w; Nrg::GFP/ UAS-trol^{DGGR201233} ; Moody-GAL4 / +
Figure 4	
(A, A')	w; Nrg::GFP / w; + / +
(B, B')	w; Nrg::GFP / w; mmp2^{W307X} / mmp2^{k00604}
(D)	w; mmp2^{W307X}, UAS-gapGFP, UAS-MoeGFP / + ; repo-GAL4 / +
(E)	w; mmp2^{W307X}, UAS-gapGFP, UAS-MoeGFP / mmp2^{k00604} ; repo-GAL4 / +
Figure S1	
(A-F")	yw ; Mmp2$^{MI00489\text{-}GFSTF.2}$
(G-H")	yw / w ; Mmp2$^{MI00489\text{-}GFSTF.2}$ / mmp2-GAL4, UAS-mCD8::mRFP
(I-L)	w ; mmp2-GAL4 / UAS-mCD8::mRFP
Figure S2	
(A)	w, Nrg::GFP / w ; mmp2^{W307X} / +
(B)	w, Nrg::GFP / w ; mmp2^{W307X} / mmp2^{k00604}
Figure S3	
(A)	w; Nrg::GFP / w; Moody-GAL4 / UAS-GFP^{S65T}
(B)	w; Nrg::GFP / w; Moody-GAL4 / + ; UAS-LanA^{DGGR207443} / +
(C)	w; Nrg::GFP / w; Moody-GAL4 / UAS-LanB1^{DGGR205294}
Figure S4	
(A)	w^{1118}
(B)	w; mmp2^{k00604} / mmp2^{k00604}
(C)	w; vkg^{ICO} / vkg^{ICO}
(D)	w; trol^{null} / w; trol^{null}
Figure S5	
(A)	w^{1118}
(B)	w; mmp2^{k00604} / mmp2^{k00604}

(C)	w; mmp1^{k04809} / mmp1 ^{k04809}
(D)	w, Nrg::GFP /w ; mmp1^{Q112X} / +
(E)	w, Nrg::GFP /w ; mmp1^{Q112X} / mmp1^{Q112X}
Figure S6	
(A)	w, Nrg::GFP / w ; Moody-GAL4 / UAS-lacZ dsRNA
(B)	w, Nrg::GFP / w ; Moody-GAL4 / + ; UAS-mmp2 dsRNA$^{NIG1794-1R-1}$ / +
(C)	w, Nrg::GFP / w ; Moody-GAL4 / UAS-mmp2 dsRNA^{BL61309}
(D)	w, Nrg::GFP / w ; Moody-GAL4 / + ; UAS-mmp2 dsRNA^{BL31371} / +
(E)	w, Nrg::GFP / w ; Moody-GAL4 / UAS-lacZ
(F)	w, Nrg::GFP / w ; Moody-GAL4 / + ; UAS-TIMP / + ;
Figure S7	
(A)	w; mmp2^{W307X} / + ; NrxIV::GFP / +
(B)	w; mmp2^{W307X} / mmp2^{k00604} ; NrxIV::GFP / +
Figure S8	w ; MZ1251-GAL4, UAS-moodyβ-GFP, mmp2 ^{k00604} / UAS-mCherry^{NLS}, mmp2 ^{W307X}
Figure S9	
(A-C)	w ; mmp2-GAL4 / UAS-mCD8::mRFP
